# Rapid High-Yield Transient Expression of Swine Hepatitis E ORF2 Capsid Proteins in *Nicotiana benthamiana* Plants and Production of Chimeric Hepatitis E Virus-Like Particles Bearing the M2e Influenza Epitope

**DOI:** 10.3390/plants9010029

**Published:** 2019-12-24

**Authors:** Gergana G. Zahmanova, Milena Mazalovska, Katerina H. Takova, Valentina T. Toneva, Ivan N. Minkov, Eugenia S. Mardanova, Nikolai V. Ravin, George P. Lomonossoff

**Affiliations:** 1 Department of Plant Physiology and Molecular Biology, University of Plovdiv, 4000 Plovdiv, Bulgaria; 2 Center of Plant System Biology and Biotechnology, 4000 Plovdiv, Bulgaria; 3 Institute of Molecular Biology and Biotechnologies, 4000 Plovdiv, Bulgari; 4 Institute of Bioengineering, Research Center of Biotechnology of the Russian Academy of Sciences, Moscow 119071, Russia; 5 Department of Biological Chemistry, John Innes Centre, Norwich NR4 7UH, UK

**Keywords:** Hepatitis E virus, chimeric HEV VLPs, Influenza A M2e

## Abstract

The Hepatitis E virus (HEV) is a causative agent of acute hepatitis, mainly transmitted by the fecal-oral route or zoonotic. Open reading frame (ORF) 2 encodes the viral capsid protein, which is essential for virion assembly, host interaction, and inducing neutralizing antibodies. In this study, we investigated whether full-length and N- and C-terminally modified versions of the capsid protein transiently expressed in *N. benthamiana* plants could assemble into highly-immunogenic, virus-like particles (VLPs). We also assessed whether such VLPs can act as a carrier of foreign immunogenic epitopes, such as the highly-conserved M2e peptide from the Influenza virus. Plant codon-optimized HEV ORF2 capsid genes were constructed in which the nucleotides coding the N-terminal, the C-terminal, or both parts of the protein were deleted. The M2e peptide was inserted into the P2 loop after the residue Gly556 of HEV ORF2 protein by gene fusion, and three different chimeric constructs were designed. Plants expressed all versions of the HEV capsid protein up to 10% of total soluble protein (TSP), including the chimeras, but only the capsid protein consisting of aa residues 110 to 610 (HEV 110–610) and chimeric M2 HEV 110–610 spontaneously assembled in higher order structures. The chimeric VLPs assembled into particles with 22–36 nm in diameter and specifically reacted with the anti-M2e antibody.

## 1. Introduction

Hepatitis E virus infection is a worldwide problem. It is thought that 50% of acute hepatitis in adults is due to Hepatitis E, with a death rate of 1–2%, rising to 20% in pregnant women [1,2]. HEV is classified in the *Hepeviridae* family, with at least four genotypes (1–4) of the virus infecting humans and animals [3,4,5]. Genotypes 1 and 2 are restricted to humans, while 3 and 4 are zoonotic and responsible for autochthonous infections in humans [3]. HEV-3 is the most common genotype detected in both humans and swine in industrialized nations [4,5]. Hepatitis E is a small, nonenveloped virus with a genome that consists of a single molecule of positive-sense RNA that contains three open reading frames (ORFs) [6]. ORF1 encodes the viral nonstructural polyprotein [7], ORF2 encodes the viral capsid protein [8], and ORF3 encodes a small regulatory protein with multiple functions [9,10]. The virion is made of 180 copies of the ORF2-encoded coat protein that is assembled to form the icosahedral shell with an approximate diameter of 27–32 nm [9]. Presently, all efforts aimed at the development of a Hepatitis E vaccine are focused on the ORF2 capsid protein, as it contains epitopes that can induce neutralizing antibodies [11,12]. The ORF2 capsid protein is also an appropriate candidate for the serological diagnosis of HEV [13]. The full-length HEV ORF2 capsid protein consists of 660 aa, with a molecular weight of 72 kDa. The protein includes an ER localization signal at its N-terminus, followed by an arginine-rich signal sequence involved in viral RNA encapsidation [14]. The capsid protein folds into three domains: S (shell; amino acids 112–319), M (middle; amino acids 320–455), and P (protruding; amino acids 456–606) [15]. The S-domain assembles into a stable icosahedral shell, while the P-domain protrudes as a surface spike, and is involved in host interactions and contains neutralization epitopes [16]. When overexpressed in mammalian and insect cells, ORF2 products with sizes between 53–88 kDa were observed [17,18]. When expressed in insect cells, ORF2 produced an insoluble 72 kDa, full-length protein and a soluble form of 56.5 kDa, consisting of a processed product [19]. Further studies in different insect cell lines showed the production of a soluble form of the ORF2 product with a molecular mass of 53 kDa that lacks the N-terminal 111 aa and C-terminal 52 aa of the ORF2 polypeptide. This retained the ability to form VLPs with T = 1 symmetry [20]. The recombinant HEV VLPs have similar antigenicity and surface structure to the wild-type virus, and elicit a strong mucosal and systemic immune response [21]. Recombinant HEV capsid protein expressed in insect cells was shown to protect primates against acute hepatitis [22], indicating this could be used for development of a recombinant HEV vaccine [23]. 

In the current study, we describe the efficient transient expression of HEV ORF2 genotype 3 capsid proteins in *N. benthamiana* using two expression vectors: the Cowpea Mosaic Virus (CPMV)-based vector pEAQ-*HT* [24,25] and the potato X virus (PVX) -based vector pEff [26]. pEAQ-*HT* is a nonreplicating system which uses the production of a highly-translatable mRNA to achieve high level expression. The recombinant vector pEAQ contains 5’-UTR and 3’-UTR from CPMV RNA-2 upstream and downstream, respectively, of the target [24]. The pEff vector is self-replicating in plant cells; it comprises the 5′-nontranslated region of the PXV genome, the gene for RNA-dependent RNA polymerase, the first promoter of subgenomic RNAs, AMV translation enhancer (5′-nontranslated region of RNA of the alfalfa mosaic virus), the *GFP* gene flanked by unique restriction sites *Asc*I and *Sma*I, the last 60 nucleotides of the coat protein gene, and the 3′-nontranslated region of the PXV genome. This construction is inserted between the 35S promoter and 35S terminator. Additionally, the vector contains an expression cassette for the silencing suppressor, P24, from grapevine leafroll-associated virus-2 [26].

Here, two expression vectors, i.e., pEAQ-*HT* and pEff, were used for the expression of ORF2 capsid constructs and chimeric constructs in *Nicotiana benthamiana* by *Agrobacterium*-mediated transient expression. The work was started with pEAQ-*HT* before the pEff system was developed. After the appearance of new, potentially more efficient system, it was used for already-selected best constructs (1) for comparison, and (2) for subsequent large-scale expression for animal studies.

To investigate the influence of the N- and C-terminal part of the HEV capsid protein on protein stability and VLP formation, we designed six constructs encoding different modifications of the N-terminal, the C-terminal, or the both parts of the HEV capsid proteins. We also investigated the feasibility of plants for the cost-effective production of HEV VLPs as candidate vaccines and as a potential scaffold for the presentation of M2e epitope from Influenza virus. We chose the exterior loop of the P domain between 539 aa and 569 aa of the capsid protein as the insertion site of the M2e foreign epitopes, because a previous study suggested that this region was permissive for manipulation [27]. We also assessed whether HEV ORF2 capsid proteins and chimeric proteins were able to self-assemble in plants into potentially strongly-immunogenic forms, such as VLPs. 

## 2. Results

### 2.1. Gene Design and Gene cloning

**Figure 1:**
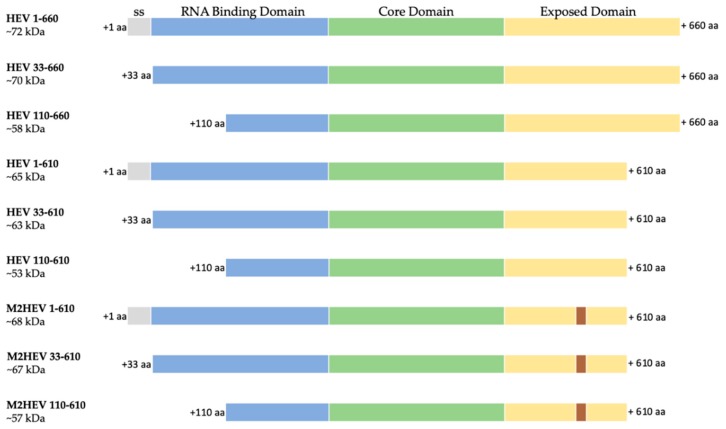
Schematic representation of the nine HEV constructs; ss–signal sequence (grey), RNA binding domain (blue), Core domain (green) and Exposed domain (yellow) containing the M2e peptide (brown) are presented as bars with different colors; 1. HEV 1–660 (full length construct); 2. HEV 33–660 (without leader sequences); 3. HEV 110–660 (N-terminal truncated); 4. HEV 1–610 (C-terminal truncated); 5. HEV 33–610 (C-terminal truncated without leader); 6. HEV 110–610 (N- and C-terminal truncated); 7. M2 HEV 1–610 (chimeric construct based on the C-terminal truncated construct); 8. M2 HEV 33–610 (chimeric construct based on the C-terminal truncated construct without leader); 9. M2 HEV 110–610 (chimeric construct based on the N- and C-terminal truncated construct).

The nucleotide sequences of the whole HEV genotype 3 nucleoprotein (HEV 1–660) and chimeric M2 HEV 1–610, codon-optimized for *N. benthamiana*, were used as templates for PCR amplification to construct the HEV 33–660, HEV 110–660, HEV 33–610, and M2 HEV 33–610. Additional constructs were designed using the M2 HEV 1–610 as a master gene and restitution with *Mun*I, and cutting the nucleotides cooding the M2e epitope (See Materials and Methods). Nine different HEV ORF2 variants (Figure 1) were cloned into pEAQ-*HT* and, after confirming the sequence of the insert, were transformed into *A. tumefaciens* and used to infiltrate *N. benthamiana* leaves.

The constructs HEV 110–610 and chimeric M2 HEV 110–610 were also cloned into the pEff expression vector, transformed into *A. tumefaciens*, and used for the expression of recombinant proteins in *N. benthamiana*.

### 2.2. Protein Expression in Nicotiana Benthamiana Plants Using pEAQ Vector

**Figure 2:**
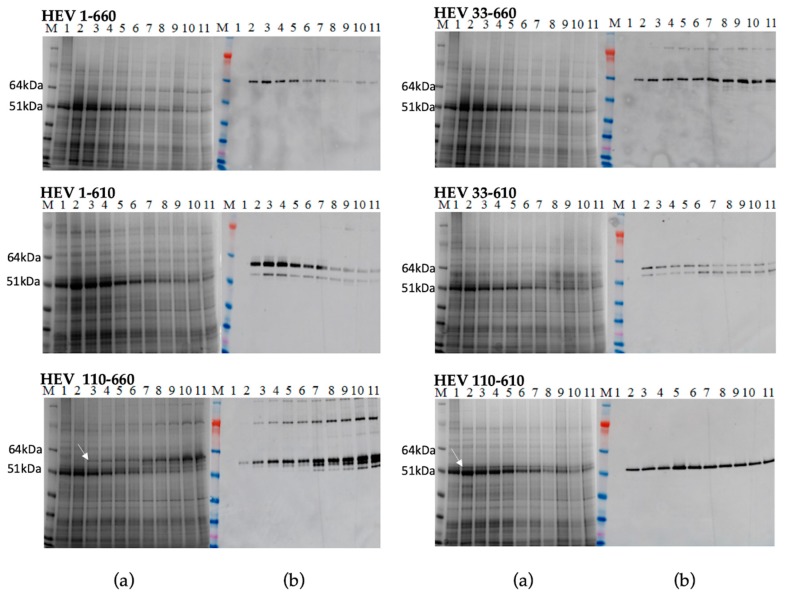
Transient expression of all constructs. Samples were collected day 2–11 post infiltration and analysed on SDS-PAGE gel (**a**) M. Molecular weight marker (kDa); 1. Leaves inoculated with the empty pEAQ-*HT* vector; 2–11 Protein samples extracted using TCA method from inoculated plant from day 2 pi to day 11 pi; (**b**) Western blot using anti-HEV ORF2 mAb, the samples are in the same order.

We produced constructs encoding various lengths of the HEV ORF2 capsid protein to analyse the effect of removing the N and/or C-terminal portions on the accumulation of the protein and its ability to form virus-like particles. *N. benthamiana* leaves were agroinfiltrated in parallel experiments with six different constructs: HEV 1–660, HEV 33–660, HEV 1–610, HEV 33–610, HEV 110–660, and HEV 110–610. At 2 to 11 days post-infiltration (dpi), samples were collected and total protein extracts prepared and subjected to SDS-PAGE (Figure 2a) analysis, followed by staining with Instant blue. An extra band, missing from the proteins extracted from leaves inoculated with the empty pEAQ-*HT* vector (Figure 2a lane 1), was visible in extracts prepared from leaves inoculated with HEV 110–660 or HEV 110–610 (white arrows). Mass spectrometry analysis confirmed that these extra bands were HEV ORF2-related proteins. By contrast, no clear additional bands were visible in samples prepared from leaves inoculated with the other four constructs, indicating that their accumulation was either lower or that the bands were masked by host proteins. Western blot analysis of extracts using an anti-HEV ORF2 mAb (ab101124) confirmed that leaves inoculated with all six constructs did, in fact, produce HEV capsid proteins, though the levels varied. Additionally, some smaller products were observed, the origins of which are discussed below. 

The expression level of the recombinant proteins and the time course accumulation of HEV ORF2 proteins were determined by Western blot and capture ELISA. The level of expression of the most HEV constructs reached their maximum on day five or six post infiltration, except HEV 110–660, which reached its maximum on day ten. We did not observe any necrosis of the leaf tissue in the infiltration zones. Hence, we determined by Western blot and capture ELISA (Figure S1, Supplementary Materials) that all constructs were expressed successfully, with the levels of expression being much higher for the constructs lacking the 110 aa from N-terminus (up to 0.8 mg/g fresh-weight tissue). 

The full-length HEV ORF2 protein product is predicted to contain 660 amino acids and to have a mass of 72 kDa. Expression of the full-length HEV 1–660 construct and the HEV 33–660 construct in plants produced only one protein in size 64 kDa. Both the 1–610 and 33–610 constructs gave products of 58 kDa and 53 kDa, while the expected sizes of the proteins were 65 kDa and 63 kDa, respectively. Expression of the HEV 110–660 construct produced several immunoreactive proteins with size 97 kDa, 58 kDa, 53 kDa, and 51 kDa. Expression of the HEV 110–610 gene gave only one protein product in size 53 kDa (Figure 2). During the expression of the different forms of the HEV ORF2 capsid gene in plants, we observed that plant cells expressed ORF2 capsid proteins and cleaved the proteins to smaller forms, as previously observed in mammalian cells.

### 2.3. Purification of HEV VLPs

Five plants per construct were infiltrated with each of the six HEV constructs, and the leaf material was collected at the time of maximum expression, as determined by the time-course experiment (Figure 2). The total protein extract was fractionated into soluble and insoluble fractions and analysed by SDS-PAGE and Western blot with anti-HEV ORF2 Ab (ab101124). From the Western blot data (Figure S2, Supplementary Materials), it appears that although the constructs HEV 1–660, HEV 33–660, HEV 1–610, and HEV 33–610 all express the HEV capsid protein, most of the protein is insoluble. Even though samples were further analysed to see if the small amount of soluble protein formed VLPs, no signals on Western blot were detected after density gradient centrifugation (data not shown). 

**Figure 3:**
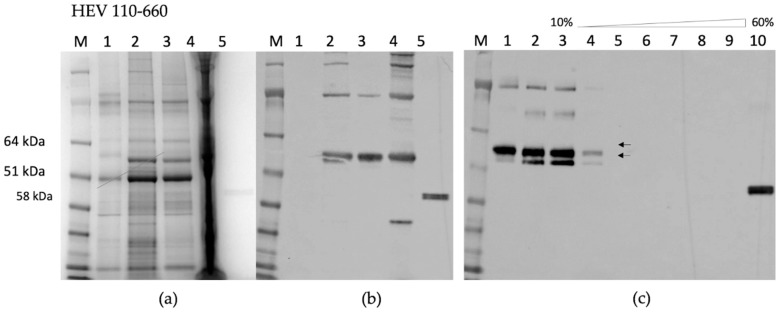
(**a**) SDS-PAGE of HEV 110–660; M. Molecular weight marker (kDa); 1. pEAQ-*HT* empty vector; 2. Crude extract; 3. SN (soluble protein) after extraction in 3× volume extraction buffer and 13k rpm; 4. Pellet (insoluble part); 5. Positive control (HEV ORF2 452–617 aa recombinant protein) (**b**) Western blot with anti-HEV ORF2 mAb, the samples are in the same order; (**c**) Sucrose gradient; M. Molecular weight marker (kDa); 1. SN after pelleting; 2. Dissolved pellet from gradient; 3. 10% sucrose; 4. 20%; 5. 30%; 6. 40%; 7. 50%; 8. 60%; 9. Pellet; 10. Positive control (HEV OF2 452–617 aa recombinant protein).

By contrast, HEV 110–660 was expressed highly in plant tissue, and is mostly soluble (Figure 3a). As found in the time course, it mainly consisted of products of 97 kDa, 58 kDa, and 53 kDa (Figure 3b). The protein mainly remains at the top of the sucrose gradient after centrifugation, suggesting that it does not assemble into VLPs (Figure 3c). 

**Figure 4:**
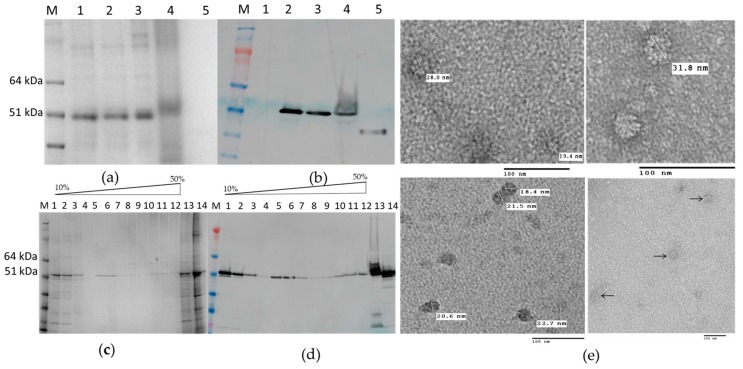
(**a**) SDS-PAGE gel and (**b**) Western blot of HEV 110–610 with anti-HEV ORF2 mAb; M. Molecular weight marker (kDa); 1. pEAQ-*HT* empty vector; 2. Crude extract; 3. SN (soluble protein) after extraction and spin; 4. Pellet-Insoluble part; 5. Positive control (HEV ORF2 452–617 aa recombinant protein); (**c**) SDS-PAGE of sucrose gradient fractions and (**d**) Western blot of fractions using anti-HEV ORF2 mAb; M. Molecular weight marker (kDa); 1 to 12 sucrose fractions from 10% to 60%; 13. Pellet; 14. SN after pelleting; (**e**) Electron microscopy of 30% (*w/v*) fraction and pellet.

HEV 110–610 expression gave a single band with 53 kDa molecular mass; like HEV 110–660, this material is mostly soluble (Figure 4a). Furthermore, some of the protein migrated down a sucrose gradient after centrifugation, as judged by Western blot analysis of the fractions (Figure 4b), suggesting that it is capable of forming higher order structures, such as VLPs. To confirm that the protein detected in the 30% sucrose gradient fraction and pellet was in the form of VLPs, samples were dialyzed, concentrated, and visualized by transmission electron microscopy. The samples of 30% and pellets both contained higher-order structures (Figure 4e).

The N-terminal and C-terminal truncated HEV 110–610 capsid protein was capable of assembling in highly-ordered structures. We observed structures with diameters varying from 19 nm to 31 nm by TEM using negative staining. Analysis of the material by cryo EM also suggested that the material was heterogeneous (M. Byrne, University of Leeds, personal communication). The fact that the HEV 110–610 protein is spread over several sucrose fractions also suggests that VLPs have heterogeneous structures.

### 2.4. Expression Using pEAQ Vector and Purification of the Chimeric Proteins

**Figure 5:**
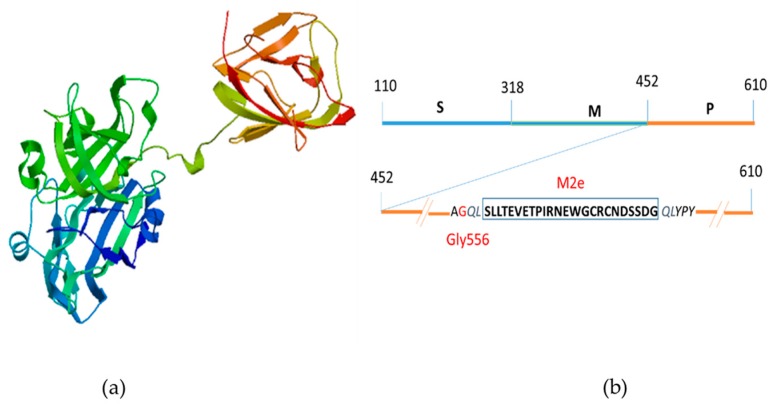
Schematic diagram of the chimeric M2 HEV ORF 2 capsid protein; (**a**) 3D modeling of M2 HEV ORF 2 capsid protein by SWISS MODEL; (**b**) Insertion of 24 amino acid residues of Influenza A virus M2 peptide at the position Gly556 (red) on the P domain of HEV ORF2 capsid protein.

The P domain of HEV ORF2 capsid protein forms a β-barrel, consisting of nine antiparallel β-strands running from one end of the molecule to the other. There are three loops, which connect adjacent β-strands, including the following positions in the sequence: 480–490, 550–565, and 580–590. These three regions have been determined to be the most suitable sites for peptide insertion [28]. Based on our previous study [27], we constructed a fusion protein (Figure 5a) by inserting the 24 aa M2e antigenic peptide from the influenza A virus after residue Gly556 into the P domain of HEV (Figure 5b).

The chimeric constructs (M2 HEV 1–610, M2 HEV 33–610, and M2 HEV 110–610; see Figure 1) were created and expressed in plants parallel with the HEV constructs. Inoculated plants produced all chimeric proteins, but only M2 HEV 110–610 accumulated in large quantities. The expression pattern of the chimeric proteins mirrored the expression pattern of their parent constructs (Figure S3, Supplementary Materials). Mass spectrometry additionally confirmed that the band with a molecular mass around 56 kDa (Figure S3a, Supplementary Materials) was the chimeric M2 HEV 110–610 protein. The *N. benthamiana* leaves infiltrated with M2 HEV 1–610, M2 HEV 33–610, or M2 HEV 110–610 were harvested on day six pi and recombinant proteins were purified by sucrose gradient centrifugation. The recombinant proteins M2 HEV 1–610 and M2 HEV 33–610 were mainly in upper fractions; however, no chimeric VLPs were observed from these constructs.

### 2.5. Self-Assembly of the Chimeric M2 HEV 110–610 into VLPs

**Figure 6:**
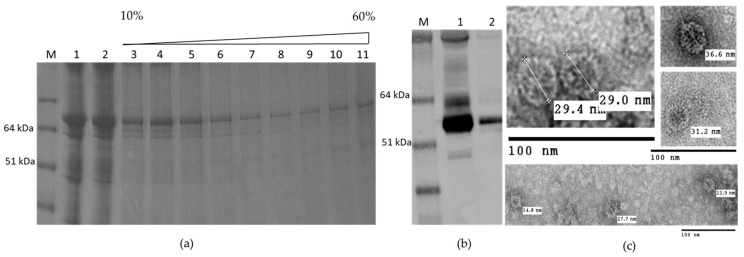
(**a**) SDS-PAGE of sucrose gradient of M2 HEV 110–610; M. Molecular weight marker (kDa); 1. TSP; 2. SN after spin the gradient; 3–10. Different sucrose fractions; 11. Pellet; (**b**) Western blot with anti-HEV ORF2 mAb; M. Molecular weight marker (kDa); 1. M2 HEV 110–610 supernatant 2. Fractions four to seven first were pooled and then sedimented by ultracentrifugation; (**c**) TEM of M2 HEV 110–610 after deliberate pelleting.

We attempted to purify the chimeric VLPs from leaves inoculated with the M2 HEV 110–610 construct using the protocols described in Material and Methods. The M2 110–610 protein migrated through all sucrose gradient fractions after centrifugation (Figure 6a), suggesting that VLPs were heterogeneous. By electron microscopic observation of the pelleted gradient fractions (Figure 6b), the chimeric M2 HEV 110–610 VLPs were visualized. The particles had a diameter from 22 nm to 36 nm. M2 HEV 110–610 VLPs were slightly bigger and more abundant than HEV 110–610 VLPs without the M2e peptide (Figure 4e and Figure 6c).

**Figure 7:**
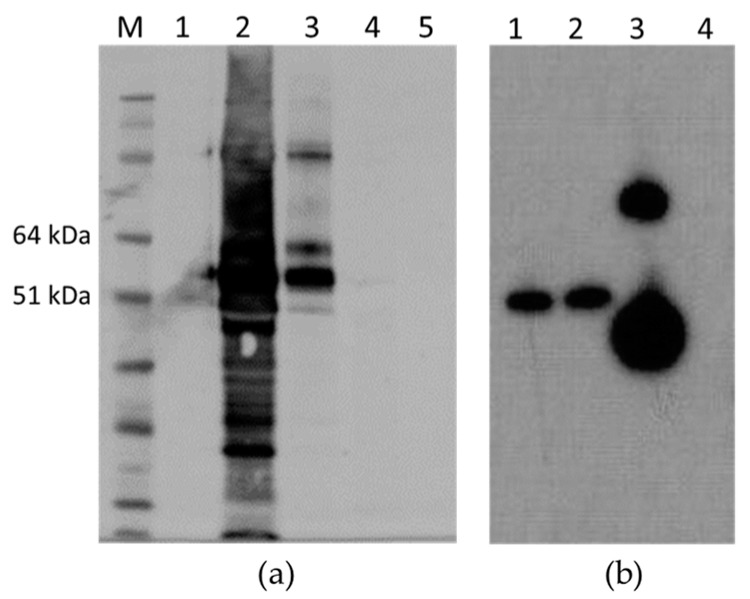
(**a**) Western blot with anti-M2e mAb; M. Molecular weight marker (kDa); 1. Empty pEAQ-*HT*; 2. TSP extracted from M2 HEV 110–610; 3. Gradient purified M2 HEV 110–610 protein; 4. TSP extracted from HEV 110–610 crude extract; 5. HEV 110–610 after purification; (**b**). Western blot with anti-M2e mAb of TSP purified from different chimeric constructs; 1. M2 HEV 33–610; 2. M2 HEV 1–610; 3. M2 HEV 110–610 and 4. Empty pEAQ-*HT*.

The antigenicity of the M2e peptide was maintained in all cases, i.e., M2 HEV 1–610, M2 HEV 33–610, and M2 110–610; Western blot with anti-M2 antibody resulted in a very strong recognition of the M2 HEV 110–610 chimeric protein (Figure 7). 

### 2.6. Protein Expression in Nicotiana Benthamiana Plants Using pEff Vector

**Figure 8:**
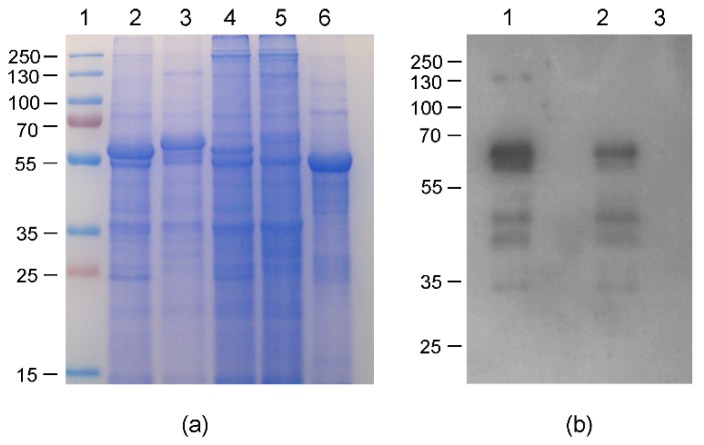
Relative efficiencies of PVX- and CPMV-based vectors; (**a**) Coomassie brilliant blue stained gel of 1.5 mg fresh weight (FW) and (**b**) Western blot of proteins isolated form *N. benthamiana* plants on day 4 post infiltration and separated by SDS-PAGE and analysed with anti-M2e Ab; (**a**) 1. Molecular weight marker (kDa); lanes 2–5, total soluble proteins isolated from zones of leaves infiltrated with agrobacteria carrying recombinant vectors pEff-HEV 110–610 (lane 2), pEff-M2 HEV 110–610 (lane 3), pEAQ-HEV 110–610 (lane 4) and pEAQ-M2 HEV 110–610 (lane 5). Lane 6 total soluble proteins isolated from noninfiltrated zone; (**b**) Lanes 1–3, total soluble proteins isolated from zones of leaves infiltrated with agrobacteria carrying recombinant vector pEff-M2 HEV 110–610 (lane 1), pEAQ-M2 HEV 110–610 (lane 2) and total soluble proteins isolated from noninfiltrated zone (lane 3).

According to our data, the constructs HEV 110–610 and chimeric M2 HEV 110–610 were chosen for a performance comparison of the pEAQ and pEff expression systems. The corresponding recombinant vectors were introduced into *A. tumefaciens* strain GV3101, which was used for agroinfiltration of leaves of *N. benthamiana* plants. Agroinfiltration zones for all four recombinant vectors were located within one leaf. Protein samples were isolated in two, three, four, five, and six days post infiltration and analysed using SDS-PAGE. In the case of pEff, the level of expression of HEV 110–610 reached its maximum on day four post infiltration, while pronounced necrosis of the leaf tissue in the infiltration zones occurred later. Summarizing the expression results for several leaves, we found that the pEff construct provides higher expression levels in comparison to pEAQ, especially for the chimeric protein M2 HEV 110–610 (SDS-PAGE results shown in Figure 8a). HEV 110–610 and M2 HEV 110–610 were highly expressed in the pEff system at 4 dpi, and accounted for about 10% of the soluble protein fraction (Figure 8). The recombinant protein M2 HEV 110–610 was revealed in Western blot analysis with the monoclonal antibodies specific to M2e (Figure 8b).

**Figure 9:**
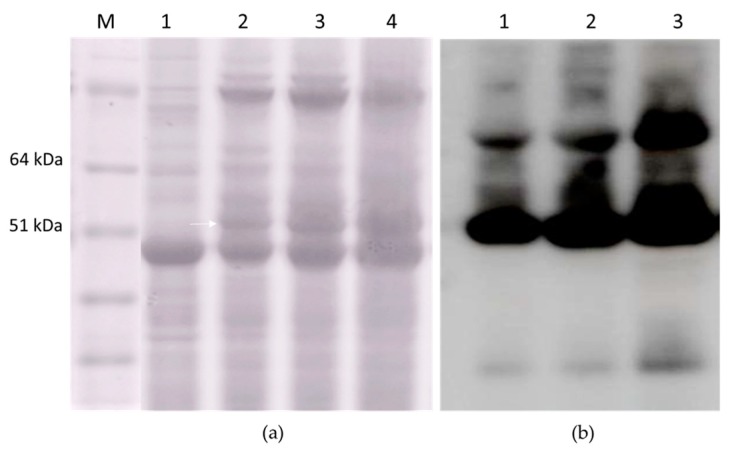
(**a**) SDS-PAGE of 3 mg FW; M. Molecular weight marker (kD); 1. Plant inoculated with empty pEAQ-*HT*; 2. TSP extracted at two dpi; 3. TSP extracted at four dpi; 4. TSP extracted at six dpi; and (**b**) Western blot with anti-HEV ORF2 mAb of TSP extracted from M2 HEV 110–610 at two dpi (lane 1), at four dpi (lane 2), and at six dpi (lane 3).

The transient overexpression of the HEV 110–610 and M2 HEV 110–610 with pEff vector induced necrosis in *N. benthamiana.* Five to six dpi necrosis appeared in the infiltrated area of the leaves with the pEff vector. In contrast, the infiltrated leaves with the HEV ORF2 constructs and the chimeric construct in the pEAQ-*HT* vector, in all cases, did not show any sign of necrosis, and in some cases (HEV 110–660), the expression reached its maximum on day ten pi (Figure 2). Compared with pEff vector, the pEAQ-*HT* vector reached the higher expression after five dpi, especially for the M2 HEV 110–610 chimeric protein (Figure 9).

## 3. Discussion

Virus-like particles have gained increasing interest in vaccine development due to their repetitive antigenic structures which have been shown to act as strong immunogens that are capable of activating both humoral and cellular immune responses without the need for any adjuvants [29]. We report here the expression and the self-assembly of HEV VLPs and chimeric HEV VLPs bearing the M2e influenza epitope. This research represents the first report of the successful transient expression of HEV VLPs and chimeric HEV VLPs in plants. This study further affirms the suitability of HEV virus-like particles as scaffolds of foreign epitopes and their possible application for the generation of monovalent or multivalent vaccines. Here, however, we observed that VLPs that expressed in plants have a heterogeneous structure and aggregation-prone particles (Figure S4). A similar phenomenon was observer when PEMV1 CP was expressed in plants [30].

Our goal was to determine whether the Hepatitis E ORF2 capsid protein could be transiently produced in plant leaves, and ultimately, whether it assembles into virus-like particles. For the production of VLPs, the full-length gt3 HEV ORF2 and ORF2 capsid protein modified at both the N- and/or C-termini (Figure 1) were expressed in *N. benthamiana* plants using the pEAQ-*HT* transient expression system. Western blot analysis of leaf extracts showed that all constructs were expressed, though the levels varied. Some proteolytic products of lower molecular weight were also observed when analysing the plant material. In addition, it showed that the expression of the whole protein, and the whole protein lacking the signal sequence resulted in a protein that is not soluble. Similar results have been observed when the whole HEV ORF2 protein is expressed in insect cells [31].

The mechanisms involved in the HEV virion assembly are not clear. It was reported that in mammalian culture cells and samples from patients, HEV produced three forms of the ORF2 capsid protein: infectious/intracellular ORF2 (ORF2i), glycosylated ORF2 (ORF2g), and cleaved ORF2 (ORF2c). ORF2g and ORF2c were the most abundant antigens detected in sera from patients [32]. The ORF2i protein is associated with infectious particles. On the other hand, the full-length ORF2 capsid protein expression in insect cells resulted in various sizes of proteins with cleavages at both the N- and the C-termini [33]. Previously, when an N-terminal 111 aa-truncated ORF2 protein was expressed in Tn5 and Sf9 cells, two major peptides, i.e., 58 and 53 kDa, were generated in both cases, and only the 53 kDa protein generated in Tn5 cells self-assembled into VLPs [34]. Additionally, the expression of a version of the capsid protein consisting of aa residues 112 to 608 formed VLPs in both Tn5 and Sf9 cells, suggesting that particle formation is dependent on the modification process of the ORF2 protein. In this report, the expression of HEV 110–660 in plant resulted in the production of three protein bands which were reactive with anti-HEV ORF2 mAb, 97 kDa, 58 kDa, and 53 kDa. The size of higher molecular weight band at 97 kDa is consistent with representing dimers of the protein. The presence of the other two observed bands (58 kDa and 53 kDa proteins) suggests the presence of the same proteolytic cleavages in the HEV ORF2 capsid protein as in the insect cells. However, VLP formation was not observed, even though the protein was expressed in abundance. 

During the expression of N- and C-end truncated recombinant protein HEV 110–610, we observed the formation of potential VLPs with various sizes, i.e., from 19 nm to 31 nm. From all the other HEV ORF2 constructs, we did not succeed in purifying and observing the particles. From this study, we concluded that N-terminal truncation to aa residue 110- and C-terminal truncation to aa residue 610 is essential for HEV VLPs formation in plants. The efficiency of VLPs isolation through gradient centrifugation is very low, and we will look for other methods for the purification of the particles from plants. 

The production of Hepatitis E VLPs in plants depends on the truncation of the ORF 2 capsid protein (N-terminal 109 aa and C-terminal 50 aa), resulting in the expression of recombinant protein with molecular mass 53 kDa. The insertion of the M2e peptide into the P domain after Gly565 appeared to increase the efficiency of particle formation and the size of the particles; we observed a slightly larger size (22–36 nm) and a greater abundance of chimeric VLPs. Jutras et al., 2015, reported that co-expression of influenza M2 ion channel protein with the other recombinant protein in plants improves the stability and accumulation of the recombinant proteins [35]. We supposed that the insertion of the external domain of M2 protein stabilizes the structure of the chimeric M2 HEV 110–610 protein by conformational change. 

Although plants have been used as a host for expression of recombinant proteins for a long time, and a number of expression systems have been developed (see [36] for review), there are no universal approaches ensuring high-level expression for any protein of interest. Therefore, we compared the performance of two expression systems, i.e., the -pEAQ vector based on genetic elements of the CPMV genome, which we were unable to replicate in plant cells, and PVX-based self-replicating pEff vector. Both systems have been successfully used for the expression of a number of recombinant proteins in plants [37,38,39,40,41]. The first two–four days post infiltration of the plant leaves with pEff vector showed an accumulation of higher level of the HEV ORF2 proteins compared with pEAQ-*HT* vector expression of the same proteins. The pEAQ-*HT* vector gave higher expression after five dpi especially for the M2 HEV 110–610 chimeric protein (Figure 9). The higher expression level in the beginning (2–4 dpi) correlated with necrosis of the plant leaf tissue at extended times, which was not observed in the case of pEAQ-*HT*.

In conclusion, we reported the formation of HEV-VLPs and chimeric VLPs only after truncation of the HEV ORF2 capsid protein. The truncation of the ORF2 gene apparently allowed us to process more efficiently the capsid proteins during the transient expression into plants. Our efforts to purify HEV-VLPs and chimeric M2 HEV-VLPs, and to evaluate their immunogenic properties, are presently underway. 

## 4. Materials and Methods

### 4.1. Gene Synthesis, Cloning and Plasmid Construction

The nucleotide sequences of the whole HEV genotype 3 nucleoprotein HEV ORF2 1–660 and chimeric M2 HEV 1–610 (lacking the 50 amino acids (aa) from the C-terminus) (GenBank accession number DQ079627.1) were synthesized by Life technologies, (Carlsbad, CA, USA). These genes were used as master genes to design six HEV genes with different lengths of ORF2 capsid proteins and three chimeric genes bearing the influenza M2e peptide (see Figure 1). The M2 HEV 1–610 master gene has an *Age*I restriction site at position 333–336 bp, and was used to generate the M2 HEV 110–610 construct. Additionally, more restriction sites were added from both sides of the M2e sequence (1675–1680 bp and 1753–1758 bp *Mfe*I), allowing the production of the HEV 1–610 and the HEV 110–610 versions of the gene without the M2e epitope to occur. Amplification of DNA fragments for the production of the HEV 33–660, HEV 110–660, and HEV 33–610 constructs was carried out by PCR. All primers used for cloning are listed in Table 1. 

The PCR fragments were flanked by *Age*I and *Xho*I restriction sites (New England Biolabs, Ipswich, MA, USA), which were used for the cloning of PCR fragments into the pEAQ-*HT* vector digested with the same enzymes. The constructs HEV 1–660, HEV 1–610, and HEV 110–610 were cloned into pEAQ-*HT* from the master genes using the restriction sites *Age*I/*Xho*I, *Nru*I/ *Xho*I, and *Age*I/*Xho*I respectively; *E. coli* XL1 blue strain was used for all cloning experiments [42]. Finally, the plasmid DNA was purified and the sequence was confirmed by PCR and DNA sequencing. 

For the cloning of the HEV 110–610 and M2 HEV 110–610 constructs in the pEff vector, the corresponding sequences were amplified by PCR using primers HEV110_Asc-F/ HEV_Sma-R, and cloned into pEff vector at *Asc*I and *Sma*I restriction sites. The corresponding recombinant pEAQ vectors were used as templates.

All pEAQ and pEff recombinant vectors were transformed into the electrocompetent *A. tumefaciens* strain LBA4404 or GV3101 [43].

### 4.2. Agroinfiltration

*Agrobacterium tumefaciens* LBA4404 or GV3101 carrying the recombinant pEAQ-*HT* and pEff vectors were grown for two days at 28 °C in Luria-Bertani (LB) medium containing kanamycin (50 µg/mL) and rifampicin (50 µg/mL), and subsequently pelleted by centrifugation. Following resuspension in 10 mM MES 2-(N-morpholino) ethansulfonic acid, pH5.6, 10 mM MgCl2 and 100 µM acetosyringone to an OD600 of 0.3 and incubation of 3 h at room temperature, bacterial suspensions were syringe-infiltrated into the leaves of four–five-week old *N. benthamiana* plants. 

### 4.3. Protein Extraction, SDS-PAGE and Western Blot Analyses

To examine the expression levels of the various HEV ORF 2 and M2 HEV constructs cloned in pEAQ vector, small-scale protein extracts were prepared from leaf material harvested at different days post-infiltration (dpi). Small-scale sampling was done by excising one leaf disc from the plant and processed using a Trichloroacetic acid (TCA) method of Wu and Wang [44]. To analyse the total soluble proteins, leaf discs (100 mg) were disrupted using a FastPrep (MP Biomedicals) with 270 µL of lysis buffer [50 mM Tris-HCl pH 8.0, 120 mM NaCl, 1 mM EDTA, 0.75% (*w/v*) sodium deoxycholate, 1 mM DTT plus complete protease inhibitor cocktail (Roche)]. Leaves and lysis buffer were mixed completely by bead beating and incubated on ice for 20 min. The samples were spun at 13,000 g for 10 min, and the supernatant was used as a soluble protein extract at a concentration of 0.3 mg fresh weight (FW)/µL. SDS-PAGE was carried out using NuPAGE Bis-Tris Mini gels of 4–12% (*w/v*) or 12% (*w/v*) acrylamide (Invitrogen, Carlsbad, CA, USA); protein prestained standard SeeBluePlus 2 (Invitrogen, Carlsbad, CA, USA) was used as a size marker throughout the experiments. To load a protein extract from 3 mg FW, 10 µL of sample solution was applied to an SDS-PAGE gel. Protein bands in the gels were visualized by Instant Blue (Expedeon, Cambridge, UK) staining. For the Western blot assay, the proteins were transferred from the SDS-PAGE gel onto a nitrocellulose membrane (Bio-Rad Laboratories Ltd, Hertfordshire, UK). Membranes were blocked with 5% (*w/v*) nonfat dried milk in PBS with 0.05% Tween-20 (*v/v*) (PBST) and incubated for 1 h with mouse anti-HEV ORF2 antigen primary antibody (ab101124; Abcam, Cambridge, UK), diluted 1:1000 at room temperature, and washed with PBST. The bound antibody was detected with secondary antimouse antibody-horseradish peroxidase (HRP) (ThermoFisher Scientific, Waltham, MA, USA) diluted 1:30,000. The emitted luminescence from the ECL detection reagents (GE Healthcare Life Sciences, Buckinghamshire, UK) was detected with the ImageQuant LAS 500 system (GE Healthcare Life Sciences, Buckinghamshire, UK). For Western blot analysis using mouse anti-Influenza A M2e mAb 14C2 (ThermoFisher Scientific, Waltham, MA, USA), the membrane was incubated with 14C2 (dilution 1:1000). 

### 4.4. Capture ELISA for the HEV ORF2 Protein Quantification

A 96-well Maxisorpmicrotitre plate (Thermo Fisher Scientific, Waltham, MA, USA) was coated with ab101124 (Abcam, Cambridge, UK), diluted 1:1000 overnight at 4 °C, washed and blocked. Plant extract (1 mg fresh weight (FW)/well) was then added and incubated for 1h at 37 °C, followed by a washing step and then incubated 1 h at 37 °C with human anti-HEV ORF2 polyclonal serum (1:1000). After further washing and incubation for 1 h at 37 °C with horse radish peroxidise, conjugated rabbit anti-human antibody HRP (ab 6759; Abcam, Cambridge, UK) diluted 1:10,000, detection was performed using 1,2-phenylenediamine dihydrochloride, SIGMA aldrich substrate. An *E. coli*-derived HEV ORF2 recombinant protein (Jena Bioscience, Jena, Germany) of known concentration were used as standards. Total soluble protein was determined for each crude leaf extract using the Bradford assay (Biorad, Hercules, CA, USA) as per the manufacturer’s instructions. To calculate the expression level of HEV ORF2 recombinant proteins, ng/mg fresh weight, we compared the absorbance obtained from the ELISA and the standard curve obtained from HEV ORF2, *E. coli* recombinant protein, ng/well (Figure S1a). The absorbance values were proportionally distributed with the amount of HEV ORF2 in the standard curve range with a squared correlation coefficient (R^2^ = 0.9827). From the standard curve, the expression levels of HEV ORF2 in each construct were calculated (Figure S1b). 

### 4.5. SDS-PAGE and Western Blot during the Protein Expression with pEAQ and pEff and Comparison

For comparison of performance of pEAQ and pEff vectors, we chose the best performing constructs, i.e., HEV 110–610 and chimeric M2 HEV 110–610. *N. benthamiana* leaves were agroinfiltrated by all tested constructs simultaneously. Four recombinant strains of *A. tumefaciens* GV3101 were infiltrated into nonoverlapping parts of a leaf using a syringe without a needle. For small-scale protein extracts, leaf discs from the infiltrated zones (~10 mg) were excised to prepare a homogenous suspension in a 50 µL extraction buffer (0.4 M sucrose, 50 mM Tris (pH 8.0), 5 mM MgCl 2, 10% glycerol, 5 mM β-mercaptoethanol). The resulting mixture was centrifuged at 14,000*g* for 15 min, and the supernatant was taken. An equal volume of 2× sample buffer was added to the supernatant. Then, 15 µL of the resulting mixture (corresponding to 1.5 mg of fresh leaf tissue) was analysed by SDS-PAGE. After electrophoresis, the gel was stained with Coomassie Brilliant Blue. The intensity of the bands in stained gels was determined using the TINA v.2.07d software (Raytest Isotopenmessgeräte GmbH, Straubengardt, Germany). For the Western blot assay, the soluble protein sample was diluted tenfold in the extraction buffer and loaded onto SDS-PAGE as described above. The proteins were transferred from the SDS-PAGE gel onto Hybond-P membrane (GE Healthcare, Marlborough, MA, USA) by electroblotting. To prevent the nonspecific binding of antibodies with the membrane, it was treated with 5% (*w/v*) solution of dry milk in TBST (150 mM NaCI, 20 mM Tris, 0.1% Tween 20, pH 8.0) buffer; then, the membrane was incubated for 1 h with mouse monoclonal antibodies specific for the M2e peptide (Anti-Influenza A Virus M2 Protein antibody [14C2] (ab5416; Abcam, Cambridge, UK) diluted 1:10,000 at room temperature and washed with PBST. The bound antibodies were detected with secondary anti-mouse antibody-horseradish peroxidase (HRP) (Promega, Madison, WI, USA) diluted 1:10,000. Specific protein complexes were detected using a Western Blot ECL Plus kit (GE Healthcare, Marlborough, MA, USA).

### 4.6. Purification of VLPs 

We used two protocols for VLPs purification: 1. Large-scale sampling was done on different days post-infiltration depending on the time-course experiment by removing any large veins and noninfiltrated tissue. The leaf tissue was homogenized in three volumes of extraction buffer (10 mM Tris-HCl pH 7.4, 120 mM NaCl plus complete protease inhibitor cocktail (Roche). Large cell debris was removed by squeezing the homogenate through one layer of Miracloth (Merck KGaA, Darmstadt, Germany), and the extracts were clarified by centrifugation at 10,000*g* for 10 min at 4 °C, loaded onto 10–60% (*w/v*) continuous sucrose gradients in a buffer (10 mM Tris-HCl pH 7.4, 120 mM NaCl) and centrifuged in a SW41Ti rotor (Beckman) at 273,800 *g* for 2.5 h at 4 °C. The gradient fractions were analysed by SDS-PAGE and Western blotting. The gradient fractions with strong signals on the Western blot were mixed and pelleted by ultracentrifugation in Surespin 630/36 (Sorvall) for 4 h. Protocol 2: The supernatant collected from clarified by centrifugation protein extract was ultracentrifuged in Surespin 630/36 (Sorvall) rotor for 4 h to pellet the virus-like particles. The pellet was resuspended in 1/10 of the volume of PBS buffer and left overnight at 4 °C. The dissolved pellet was put on top of a 10–50% (*w/v*) sucrose density gradient and centrifuged at 35,000 rpm in a TH641 (Sorvall) for 2.3 h at 4 °C. Fractions were then collected by piercing the bottom of the ultracentrifuge tube and collecting the material as it dripped out. Two different purification protocols were used to optimize the amount of particles. 

### 4.7. Mass Spectrometry 

Maldi-TOF/MS was used for identification of HEV ORF 2 proteins and M2 HEV ORF2 proteins. MS/MS analysis was performed as followed: The protein bands corresponding to the HEV ORF2 capsid proteins and chimeric proteins were excised from the SDS-PAGE gel and washed. The gel was washed twice with water, and then twice with water/ACN (1:1 *v/v*). The solvent volumes were about twice the volume of the gel. The liquid was removed, ACN was added to the gel pieces, and the mixture was left for 5 min. The liquid was removed and the gel pieces were rehydrated in 0.1 M NH4HCO3 for 5 min. ACN was added to give a 1:1 *v/v* mixture of 0.1 M NH4HCO3/CAN and left to stand for 15 min. All liquid was removed and the digestion buffer containing 25 mM NH4HCO3 and 10 ng/μL of trypsin was added. The mixture was then incubated for 4 h at 37 °C. The supernatant was recovered and the extraction was carried out with 1% TFA/ACN (1:1 *v/v*). Tryptic peptides were targeted for MALDI-TOF-MS, MS/MS and for a database search.

### 4.8. Transmission Electron Microscopy

For electron microscopy analysis, samples were adsorbed onto hexagonal, carbon–coated copper grids for 30–40 s, which were then washed by floating on water droplets. Excess fluid was removed with filter paper and the grids were negatively stained with 2% (*w/v*) uranyl acetate (UA) for 20 s. Particles were imaged using a FEI Tecnai G2 20 Twin TEM. 

## Figures and Tables

**Table 1:** List of primers used in this work.

Primer Name	Primer Sequence (5′-3′)	Purpose
HEV-leader F	AAATACCGGTAACA*ATG*GGTGGTGCTGGTGGT	HEV 33–660
HEV-leader R	AGGCCTCGAG*CTA*AGACTCTCTGGTCTTTCC	HEV 33–660
HEV_N110_F	AAATACCGGTAACA*ATG*GCTACT	HEV 110–660
HEV_N33_F	AAATTCGCGAAACA*ATG*GGTGGTGCTGGTGGT	HEV 33–610, M2 HEV 33–610
HEV_N33_R	CAATCTCGAG*CTA*AGCAAGAGCAGAGTGAGGAGCAA	HEV 33–610, M2 HEV 33–610
HEV110_Asc-F	TAGGCGCGCC*ATG*GGTATGGCTACTTCTCCTG	Cloning in pEff vector
HEV_Sma-R	ATCCCGGGCTAAGCAAGAGCAGAGTGAGGAG	Cloning in pEff vector

## Supplementary Materials

The following are available online at https://www.mdpi.com/2223-7747/9/1/29/s1, Figure S1: Capture ELISA for the HEV ORF2 protein quantification (a) Standard curve with purified rHEV ORF2 protein. The y-axis is the absorbance value. The x-axis is the value of rHEV ORF2 protein. (b) The expression level of chimeric M2 HEV and HEV ORF2 in TSP extracted from fresh plant leaves inoculated with the relevant constructs. Figure S2: Western blot with anti-HEV ORF2 mAb of the constructs; (a) HEV 110–6101; (b) HEV 33–660; (c) HEV 1–610; (d) HEV 33–610; M. Molecular weight marker (kDa); 1. pEAQ-*HT* empty vector; 2. Crude extract; 3. SN (soluble protein) after extraction in 3x volume and 13k rpm; 4. Pellet (insoluble part); 5. Positive control (HEV ORF2 452–617 aa recombinant protein). Figure S3: SDS-PAGE (a) and Western blot with anti-HEV ORF2 mAb (b) of TSP extracts (1 mg FW) of HEV chimeric constructs bearing M2e peptide and its relevant HEV construct without M2e; M. Molecular weight marker (kDa); 1. Healthy plant; 2. Empty pEAQ-*HT*; 3. M2 HEV 1–610; 4. HEV 1–610; 5. M2 HEV 33–610; 6. HEV 33–610; 7. M2 HEV 110–610; 8. HEV 110–610. Figure S4: TEM of M2 HEV 110–610 sedimented fractions.
