# Supplementary material for: Rapid High-Yield Transient Expression of Swine Hepatitis E ORF2 Capsid Proteins in Nicotiana benthamiana Plants and Production of Chimeric Hepatitis E Virus-Like Particles Bearing the M2e Influenza Epitope
Source: Plants (Basel). 2019 Dec 24;9(1):29. doi: 10.3390/plants9010029 (PMC7020208; doi:10.3390/plants9010029)
Supplement: Supplementary file 1 [file plants-09-00029-s001.pdf]

Supplementary Materials:

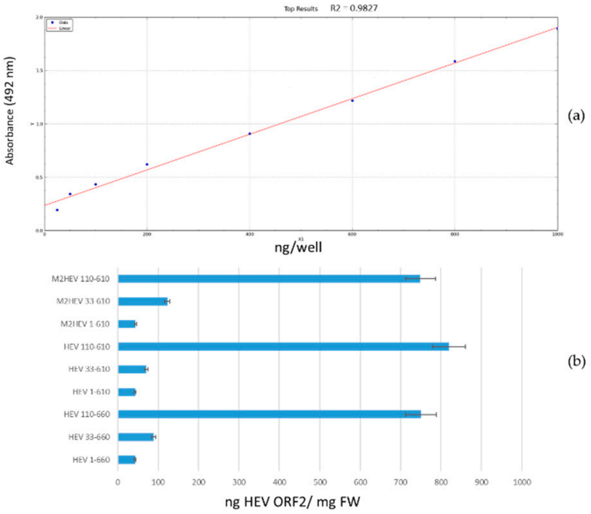

Figure S1. Capture ELISA for the HEV ORF2 protein quantification (**a**) Standard curve with purified rHEV ORF2 protein. The y-axis is the absorbance value. The x-axis is the value of rHEV ORF2 protein. (**b**) The expression level of chimeric M2 HEV and HEV ORF2 in TSP extracted from fresh plant leaves inoculated with the relevant constructs.

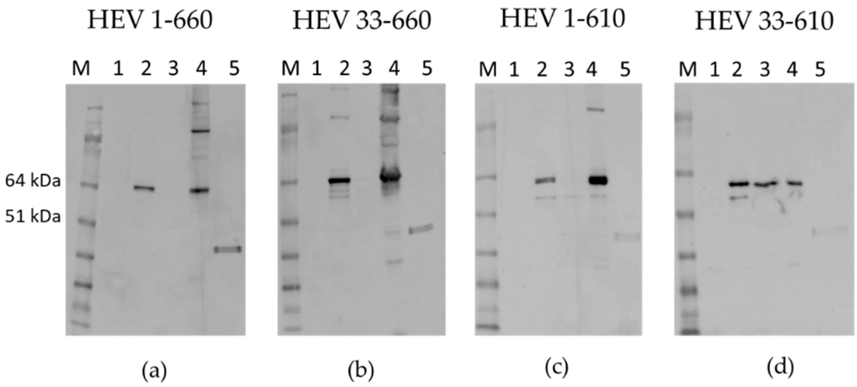

Figure S2. Western blot with anti-HEV ORF2 mAb of the constructs; (**a**) HEV 110-6101; (**b**) HEV 33-660; (**c**) HEV 1-610; (**d**) HEV 33-610; M. Molecular weight marker (kDa); 1. pEAQ-*HT* empty vector; 2. Crude extract; 3. SN (soluble protein) after extraction in 3x volume and 13k rpm; 4. Pellet (insoluble part); 5. Positive control (HEV ORF2 452-617 aa recombinant protein).

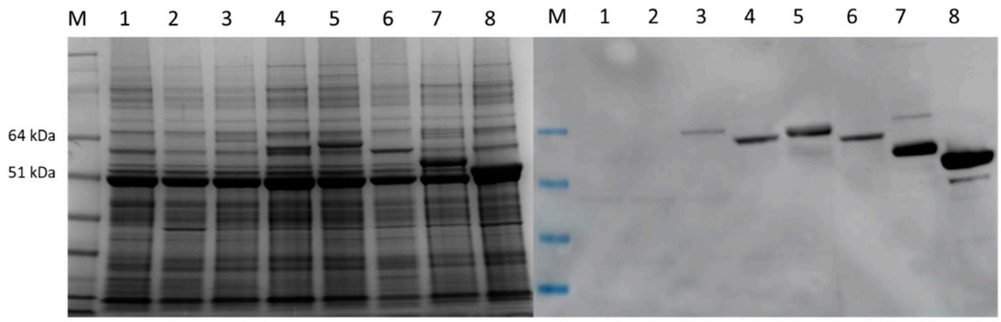

(a) (b)

Figure S3. SDS-PAGE (**a**) and Western blot with anti-HEV ORF2 mAb (**b**) of TSP extracts (1 mg FW) of HEV chimeric constructs bearing M2e peptide and its relevant HEV construct without M2e; M. Molecular weight marker (kDa); 1. Healthy plant; 2. Empty pEAQ-*HT*; 3. M2 HEV 1-610; 4. HEV 1-610; 5. M2 HEV 33-610; 6. HEV 33-610; 7. M2 HEV 110-610; 8. HEV 110-610.

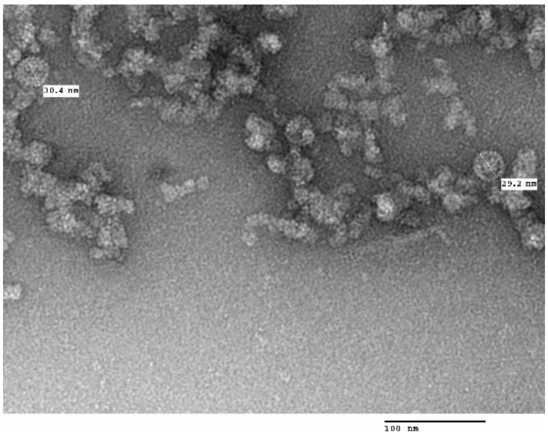

Figure S4. TEM of M2 HEV 110-610 sedimented fractions
